# Ethical considerations in surgical care: cultural and religious perspectives in Africa

**DOI:** 10.1097/MS9.0000000000003695

**Published:** 2025-08-08

**Authors:** Bisrat Abate Bekele, Charbel Kachouh, Clever Byiringiro, Abdi Degefu Gashu, Batoul Cherri, Sarah Mshaymesh, Jack Wellington, Olivier Uwishema

**Affiliations:** a Department of Research and Education, Oli Health Magazine Organization, Kigali, Rwanda; b School of Medicine, College of Health Sciences, Addis Ababa University, Addis Ababa, Ethiopia; c Department of General Dentistry, Faculty of Dental Medicine, Saint Joseph University, Beirut, Lebanon; d Department of General Medicine, Faculty of Medicine, Adventist School of Medicine of East-Central Africa, Kigali, Rwanda; e Department of Anaesthesiology and Pain Management, Faculty of Medicine, Lebanese University, Beirut, Lebanon; f Faculty of Sciences, Haigazian University, Beirut, Lebanon; g Leeds Teaching Hospitals NHS Foundation Trust, Leeds, United Kingdom

**Keywords:** Africa, cultural competence, ethics, healthcare access, informed consent, low-resource settings, patient autonomy, religious beliefs, surgical care, traditional healers

## Abstract

**Background:**

Surgical care in the continent of Africa encounters a wide array of issues, comprising a lack of access to emergency and essential surgery. Optimal pre-, peri-, and post-operative care is guided by the four pillars of medical ethics: beneficence, non-maleficence, autonomy, and justice. This may not be the case for low-resource domiciles as issues circumventing low literacy levels, poor infrastructure, poverty, and inequities in social health are cumbersome. Moreover, there is no homogeneity in the cultures and religious diversity that Africa has to offer which has led to a variety of healthcare decisions being made alongside an influence on the relationships developed between patients and their healthcare providers.

**Objectives:**

This study aims to address the influences of diverse cultural and religious backgrounds in ethical decision-making in African surgical care. Moreover, the study suggests strategies for mitigating diversity issues through developing environment-based protocols that promote ethically acceptable and culturally sensitive local surgical care.

**Methods:**

A comprehensive literature review was conducted to explore the present state of surgical care in Africa alongside the ethical principles guiding surgical care and cultural/religious considerations that may affect healthcare decisions when establishing patient-provider rapport. The databases used are PubMed/MEDLINE and EBSCOhost. Our search terms were “surgical care,” “ethical consideration,” “Africa,” and “culture and religion in Africa.” This enabled us to meticulously look into the existing findings to develop suggestions based on the gaps.

**Result:**

The literature review demonstrated the complex interplay between moral principles, cultural and religious outlooks, and surgical care provision in Africa. Some of the strategies found useful in this case are strengthening health systems, resources adequacy alongside context-based directives as well as capacity building. Additionally, cultural understanding, active listening and appreciation of diversity is critical since patients will receive culturally competent care and minimize chances for medical errors.

**Conclusion:**

Tackling Ethical challenges in African surgical care requires multiple approaches that accommodate religion’s diverse culture. To do this, healthcare workers must cooperate with community members and policymakers to develop policies for ethically and culturally acceptable surgical approaches. Further research is warranted to evaluate the efficacy of these interventions.

## Introduction

Surgical care in Africa faces many challenges including a lack of access to services due to a shortage of professionals and infrastructures, financial barriers from the high cost of surgery, and lack of prioritization due to the high burden of infectious diseases. In addition, the cultural and religious diversity in the continent greatly influences individual healthcare decisions affecting the relationships between patients and their healthcare providers. Indeed, this has been recognized by the World Health Organization (WHO) which has stressed the importance of basic surgical services coupled with meeting the needs for safe minimum facility levels as well as improvements of infrastructure within associated healthcare systems**^[1]^**. African nations have particular difficulties, such as a scarcity of surgeons and a reliance on non-physician professionals for medical treatment. Additionally, the Lancet Commission on Global Surgery emphasizes the paramount importance of district hospitals offering timely and affordable surgery**^[2]^**.

While making efforts to increase access to surgical care upholding the four major ethical precepts must be prioritized. According to these moral standards, healthcare professionals must respect the views and choices of their patients while minimize the harm inflicted. Beneficence calls for healthcare providers to retain and improve their skills and knowledge to consider the patient’s personal condition as well as strive for the welfare of the individual wholly**^[3]^**. Non-maleficence ensures that medical actions are weighed against their risks and benefits so that no treatment is regarded as a better option**^[4]^**. Autonomy recognizes the self-ownership of persons along with their prerogative of rational decision-making pertaining to morality issues concerning his or her own body**^[4]^**. Justice denotes equitable treatment without ethnic or social biases toward all patients equally deserving it**^[1]^**.

One of the ways to recognize diverse ethnic and religious outlooks is by increasing the autonomy of the patient through informed consent. Obtaining informed consent (IC) from a patient is crucial to surgical care so that they are aware of the risks, benefits, and alternatives for any given procedure**^[5,6]^**. It also improves trust between doctors and patients while at the same time shielding doctors from malpractice suits**^[7]^**. However, no formal guidelines exist about informing stakeholders concerning ethical challenges and considerations in global surgical collaborations**^[8]^**. Surgical ethics has as its conceptual framework three dimensions: actions, “good work well done,” and relationships**^[9]^**.

For instance, the principles of medical ethics were employed during the likes of the novel coronavirus disease 2019 (COVID-19) pandemic**^[10]^** and in areas depicting patient-related communication, trainee education, palliative/end-of-life care, and surgical innovation**^[11]^**. Nevertheless, applying these principles in low-resource settings is problematic due to high illiteracy rates, limited infrastructure, poverty levels, social health disparities, and a lack of political will toward equity in health systems**^[12]^**. Moreover, the diverse cultural outlook of the people makes it hard to abide by ethical principles. Religious diversity has also an effect on ethical surgical decisions. Christianity, Islam, and traditional African religions are the three most notable religious groups in Africa**^[13]^**. This diversity influences the experiences garnered by individuals with issues circumventing health and illness as well as their access to medical treatment**^[9]^**. Traditional healers play a very significant role in African society and case studies highlight how culture and religion affect healthcare choices**^[10]^**. Spiritual and religious beliefs may impact patient-provider relationships, and healthcare professionals must respect and accommodate said beliefs.

Among the techniques that may be utilized to navigate cultural and religious diversity in healthcare are that of developing an understanding of diverse cultures, employing active listening skills, and embracing cultural differences**^[14]^**. These strategies may assist in improving patient safety, reducing inefficiency and inequity in care, and promote cultural comprehension shared among healthcare multidisciplinary teams**^[15]^**.

Hence, the purpose of this study is to investigate the ethical challenges in African surgical care, taking into account cultural and religious traditions that shape decisions pertinent to the clinical management and relationships established between patients and their respective healthcare providers. Through examining these factors and identifying methods of navigating diversity, this study aimed at contributing toward the development of contextually appropriate approaches that will promote ethical as well as culturally competent surgical care within the continent.

This manuscript strictly adheres to the TITAN Guidelines 2025 for transparent declaration and use of AI in scholarly publications, and no AI tools were used in the research or manuscript preparation**^[16]^**.

**HIGHLIGHTS**

- Ethical challenges in African surgical care arise from the diverse cultural and religious beliefs that influence healthcare decisions and patient-provider relationships.

- Proposed strategies include strengthening health systems, ensuring resources adequacy, and fostering cultural understanding to improve surgical care in low-resource settings.
- Collaboration among healthcare workers, policymakers, and local communities is essential for creating sustainable and accessible surgical care policies in Africa.

## Materials and methods

This narrative review was conducted to explore the ethical challenges in surgical care across Africa, focusing on the influence of cultural and religious factors. We systematically searched the PubMed/MEDLINE and EBSCOhost databases for English-language articles published up to May 2024. Search terms included “surgical care,” “ethical consideration,” “Africa,” “culture and religion in Africa,” “informed consent,” and “cultural competence.” Relevant publications, including original research, reviews, policy documents, and case reports, were identified.

The initial search yielded 120 articles. After screening titles and abstracts for relevance, 55 full-text articles were reviewed. Studies were included if they addressed ethical issues in surgical care with a focus on Africa and discussed cultural or religious influences. Studies that focused solely on non-surgical care or non-African settings were excluded. Reference lists of selected articles were also screened for additional relevant studies.

Data from the included studies were extracted and summarized according to main themes: application of ethical principles in surgical care, cultural and religious diversity, informed consent challenges, and strategies to improve ethical practice. Insights from case studies and policy documents were used to highlight real-world examples and potential solutions.

## Ethical principles in surgical care

**Figure 1.**
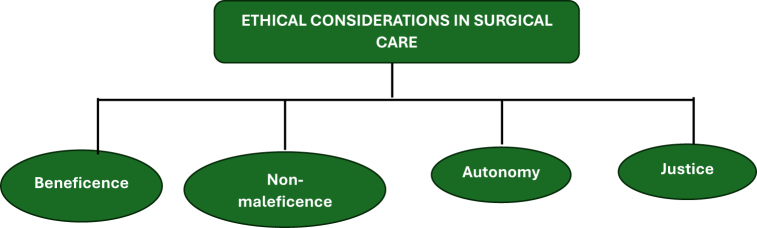
The main principles of ethics.

While giving medical care, we use ethical principles to guide the interaction between the healthcare provider and recipient in a manner that is tolerant of their cultural and religious values. The four major precepts of medical ethics are beneficence, nonmaleficence, autonomy, and justice**^[4]^**. Physicians are ethically obligated to aid patients, reduce or prevent harm, and respect their beliefs and choices despite disagreement, making ethics an essential component of clinical medicine**^[4]^** (Fig. 1).

Beneficence is described as the physician’s obligation or duty to behave in the best interests of the patient**^[4]^**. Beneficence requires healthcare personnel to improve and maintain their skills and expertise, often updating trainings, taking into account the unique circumstances of each patient, and working for the total patient’s well-being and advantages**^[3]^**.

Nonmaleficence, on the contrary, is an ethical concept that attempts to prevent patients from suffering damage. It includes moral norms such as not killing, causing pain or suffering to patients, not incapacitating, not committing a crime, and not depriving others of the necessities of life**^[4]^**. It is a notion that requires that every medical intervention be balanced against all benefits, risks, and repercussions, sometimes determining that no therapy is the best standard of care**^[17]^**.

Autonomy is an ethical principle that highlights the notion that all persons have fundamental and unconditional value, that they should be able to make logical judgments and moral choices, and that they should be permitted to exercise their right to self-determination**^[4]^**. This concept guarantees that each and every person of legal age and sound mind has the freedom to decide what to do with their own body. This simply implies that for human beings who are still minors in age and those with cognitive disabilities, autonomy ethical principle is overlooked. According to this autonomy concept, the patient must have the last say in all matters pertaining to them, and physicians must respect and act in accordance with that choice**^[18]^**.

Another ethical concept that is considered in surgical intervention is justice. It alludes to managing individuals in a just, equitable, and proper concordance**^[4]^**. According to this principle, all patients must receive the same level of care, free from bias and societal discrimination**^[19]^**. Every patient has a right to be nursed as a human being with individual demands, and the right to be cared for based on their partialities, with no consideration of one’s economic, social, or mental state, so as to best serve with fairness all patients**^[19]^**.

An important tool to uphold the above ethical principles in surgical interventions is informed consent. According to its definition, it is the concept that an individual or patient should be aware of all medical procedures that will be performed and that they should be free to choose whether or not to accept said procedures following information pertaining to the potential risks**^[20]^**. It entails informing the patient about their health status, the diagnosis made, the type of recommended treatment, its success rate, duration, and risks, as well as the administration of prescribed medication and potential adverse drug reactions, the consequences of not accepting the recommended treatment, and the risks associated with each option**^[20]^**. Additionally, it shields the doctor from any legal action as a lack of IC may place the doctor in a precarious legal situation. However, by promoting mutual understanding, IC also builds trust between the patient and the doctor**^[7]^**. Moreover, it can be used as an important tool to uphold the cultural and religious beliefs of the individuals.

The application of ethical principles in surgical care has always been observed in the context of different surgical interventions. For instance, ethics were a guiding tool for making the best available choices for patients during the COVID-19 pandemic**^[10]^**. Applying ethical concepts to patient-related communication involves issues like that of consenting, mentoring trainees, palliative and end-of-life care, and surgical innovation and research, all of which have significant effects on patients, surgeons, and society at large**^[11]^**.

However, there are difficulties when applying these principles in real life. There are currently no official rules in place to assist in educating pertinent parties about the ethical issues and obstacles that international surgical cooperation must face**^[8]^**. As surgery is a field that is action-oriented as opposed to speculation, there is a need for ethics of actions, a need for ethics of a “good work well done,” and also a need for ethics of relationship which fosters the technical mastery of surgery**^[9]^**. From this, a conceptual framework can be drawn, which considers the three axes that cover the major realities of surgery according to Anne-Laure Boch – actions, “good work well done,” and relationships**^[9]^**.

Besides, there are hindrances to the implementation of ethical principles in low-resource settings. Healthcare professionals based in low-income nations encounter several distinct ethical dilemmas in their work, the impact of which affects both patient and healthcare provider alike**^[21]^**. These difficulties are elucidated in light of several other factors, such as low literacy rates, inadequate infrastructure, poverty, disparities in social health, and the government’s scant commitment to just health systems**^[21]^**. Physicians may find it onerous to manage a patient’s divergent interests while adhering to ethical standards, given the patient’s autonomy to select familial and cultural interests and the physician’s responsibility regarding autonomy**^[21]^**. It is arduous to apply ethical principles in settings with limited resources due to the dearth of infrastructure and other medical technologies, restricting clinician autonomy in choosing when and how to treat patients**^[21]^**. In addition, low salaries may force some clinicians to employ the preferred medication and instruments posed by pharmaceutical and medical technology conglomerates.^12^ While delivering care to patients, doctors in low-income nations are limited or constrained in their capacity to apply medical ethics and, most importantly, justice**^[21]^**.

To combat the challenges of ethical principles implementation and promote ethical surgical care in Africa, strengthening health systems and infrastructure alongside allow for proper allocation of adequate resources would foster an improved surgical care**^[21]^**. To ensure that trainees engage and understand how ethical principles can be applied within their context and related dilemmas, medical education and training programs may allow students to think through various challenges they might encounter and develop context-sensitive strategies by introducing the ideal principle with supplementation of pertinent case scenarios from the local context**^[21]^**. In addition, medical schools in low-income nations should work with ethicists to create clear guidelines for practitioners. This will help to guarantee consistency in the decisions made by practitioners and might also lessen their moral distress when they are confident in the support of their peers**^[21]^**. These efforts will overall lead to more structured ethical guidance encompassing the cultural and ethical beliefs of the recipients of surgical care.

## Cultural and religious landscape in Africa

### Overview of major religious in Africa and their core beliefs

**Figure 2.**
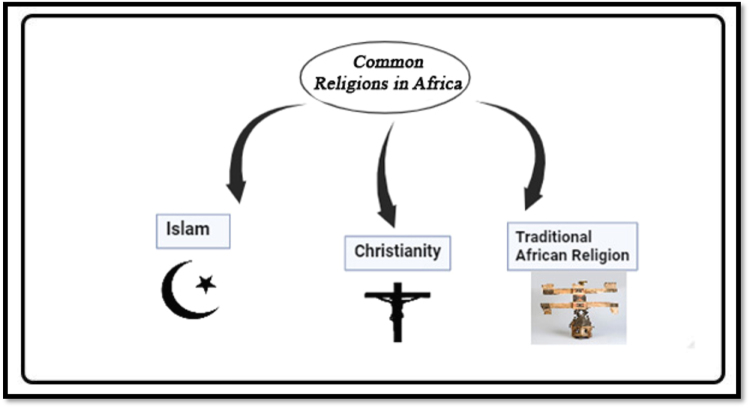
African religions.

The two introduced religions that currently dominate the continent are Islam and Christianity, but traditional faiths continue to be significant, particularly in the interior of sub-Saharan Africa (SSA)**^[13]^** (Fig. 2).

#### Christianity

In SSA, Christianity is the most populous and is practiced by more individuals than that of Islam. Across most of the continent, a number of syncretistic and messianic cults have emerged, such as the Aladura churches in Nigeria and the Nazareth Baptist Church in South Africa**^[22]^**. The oldest Christian denominations in Africa are the Ethiopian Orthodox Tewahedo Church, the Eritrean Orthodox Tewahedo Church, the Eastern Orthodox Church of Alexandria, and the Coptic Orthodox Church of Alexandria**^[23]^**.

#### Islam

Along with Christianity, Islam is the second most dominant religion in Africa, where it is practiced by over 40% of the populace, or an approximated one-fourth of all Muslims worldwide. The historical origins of the faith on the continent date back to the Prophet Muhammad’s period, when his early followers fled to Abyssinia (hijra) to avoid persecution by the pagan Arabs**^[24]^**. Islam was introduced to West Africa by Islamic traders and seamen. Islam is the most popular religion in the North and Horn of Africa**^[24]^**.

#### Traditional African religions

Africans adopt a wide range of ethnic faiths, among other beliefs and customs. These customs are transmitted orally from generation to generation through folktales, songs, and festivals. These encompass the worship of the deceased, the use of witchcraft and traditional African medicine, and the conviction that ghosts and both higher and lower gods – occasionally even a supreme being – exist**^[25]^**. Most religions can be classified as animistic because they all have various polytheistic and pantheistic components. In the minds of most, humanity’s role is to maintain a balance between nature and the supernatural**^[25]^**.

### Cultural diversity in Africa and its impact on healthcare

People’s experiences with health and sickness, as well as their access to and use of medical treatment, are significantly influenced by their culture. It alters the healing connection and presents both opportunities and obstacles for improved health outcomes. It affects healthcare personnel as well as their patients and the communities**^[26]^**.

In mainstream healthcare literature, the challenges of providing appropriate healthcare to culturally diverse populations are often ascribed to the cultural needs and issues of the patients. However, the cultures of the practitioners and the health systems are as much at play in this context as the cultures of the patients. How the cultures of both the client and the clinician/therapist impact the therapeutic alliance or relationship are very significant considerations in working with culturally and linguistically diverse cultures. The best possible situation would be where the healthcare professional and the client are from the same culture and have a shared cultural understanding. But even in these circumstances, the healthcare profession brings with them a “professional” culture that can prove a barrier to working with their clients.**^[15]^**

The lack of effective communication might lead to the patients feeling unheard and unsatisfied while causing the healthcare providers to lose important information that might help them treat their patients.

At the healthcare level, there is underrepresentation of cultural, gender, and ethnic diversity during training and in leadership. To serve the needs of a diverse population, the healthcare system must take measures to improve cultural competence, as well as racial and ethnic diversity**^[26]^**

### Role of traditional healers in African communities

Traditional healing is defined differently by varying communities. Traditional medicine is defined by the WHO as “wellness procedures, techniques, expertise, and religions including plant, animal, and mineral based medication, religious treatments, manual procedures and activity, used individually or in conjunction, in order to treat, diagnose, and avoid diseases or maintain mental health” as well as “the totality of every piece of information and practice, if comprehensible or not, utilized for identifying, avoiding, or curing a physical, psychological, or societal imbalance that rely purely on previous knowledge and experience passed handed down from family to family, either orally or in written work.”**^[27]^**

Furthermore, herbal medicine and spiritual therapy are both included in traditional healing. It takes a comprehensive approach and embodies the knowledge that indigenous people have passed down over many centuries as a collective. Even though academics use the term “traditional healing,” it may allude to a plethora of treatment philosophies that are distinct from the Western healing paradigm, traditional medicines are dynamic and varied around the world because of the diversity of their geographic origins, nationalities, and agricultural systems**^[28]^**.

### Case studies illustrating the influence of culture and religion on healthcare decisions

Culture has an impact on biomedical care in Africa because of differences in the definition of a disease, lack of health-related knowledge, poverty, and ignorance. Efforts to give ethically abiding surgical interventions are continuously affected by the deeply rooted cultural and religious beliefs in the African community. Thus, focus must be placed on health outreach initiatives, outreach programs, and campaigns across Africa, especially in the more vulnerable rural areas where these diseases are most prevalent**^[1]^**.

Additionally, we can use a more tolerant medical practice approach. Conventional medicine (CM) operates as “biological garages” that are inherently interested in the biomedical indicators of disease and illness. This is due to the influence of the classic biomedical model (BMM) on CM. However, the new Biopsychosocial-Spiritual Model recognizes that patients may have subjective experiences that are not explained by medicine and that affect how they perceive and react to treatment regimens. The BPSSM finds it difficult to get widespread acceptance and recognition by CM in Africa, despite striking a profound chord with African Traditional Medicine (ATM). Purposive therapeutic partnership between ATM and CM is necessary given the persistence of multi-health seeking behavior in Africa.**^[29]^**

### Cultural and religious factors influencing patient-provider relationships

As discussed above, majority of patients who warrant healthcare make their tough healthcare decisions based on their spiritual and religious beliefs. Many patients will turn to their faiths to help them feel less anxious when dealing with medical issues. Regrettably, physicians might fail to recognize this. The current division between religion and medicine is a result of the scientific approach to addressing medical conditions**^[30]^**.

To increase the quality of surgical care, healthcare providers should honor the importance of religious and cultural beliefs. Healthcare professionals should provide patients an opportunity to discuss their spiritual and religious views and tailor their evaluation and treatment to meet each patient’s specific needs**^[31]^**.

### Strategies for navigating cultural and religious diversity in healthcare

Cultural and religious tolerance increases the quality of healthcare, especially surgical. More patient involvement and engagement lead to increased respect and better understanding, which can enhance patient safety and reduce inefficiencies, inequities in care, and costs**^[13]^**. Improving cross-cultural communication can be achieved by assembling groups of medical professionals who are representative of the patient communities they serve. Teams made up of people with diverse origins can help each other become more culturally sensitive. They are therefore more inclined to react to patients’ particular cultural demands with empathy**^[14]^**.

Promoting understanding and training people are necessary for improving cultural competence in healthcare. Healthcare workers must become aware of their own ideas and cultivate a sense of cultural awareness to become culturally competent. They may then expand on this to improve their understanding of different cultures**^[15]^** (Table1).

**Table 1:** Strategies for navigating cultural and religious diversity in healthcare

Strategies	Effect on healthcare
Develop cultural awareness	Patient safety and reduce inefficiencies
Practice active listening	Reduce costs and inequities in care
Embrace cultural differences	Share cultural knowledge

## Ethical challenges in surgical care in Africa

IC is the ultimate choice to inform the details of a procedure to a patient. The patient must be capable of making personal decisions about whether to undergo a procedure or not**^[32]^**. IC may be presented in various manners and methods using different types of consent forms. It is highly affected by the interaction between patient and health care provider. Thus, Difficulties in understanding, compliances, and voluntariness for clinical trials are leading drawbacks for IC assessment**^[6]^**. To improve ethical and legal standards, persistent and continuous efforts are required to reveal the practical realities of IC**^[5]^**.

While making this consent, Illiteracy and language could lead to barriers to communication which prevent basic understanding of medical procedures, by that means if illiterate patients are incapable of understanding IC, this could increase medical errors, bereaving patients of their constitutional right to access information therefore leading to medical misconduct**^[12]^**. Due to variety of languages, communication barriers between clinicians and patients could arise thus causing difficulties in patients care. This can be problematic and frustrating for both healthcare providers and patients**^[33]^**. The main dilemma remains in obtaining IC for invasive inpatient interventions for patients with Limited English Proficiency (LEP). To counteract this issue and due to the limited in-person availability of professional translators and interpreters in most hospitals, patients often refer to other bilingual family members to interpret**^[34]^**.

Inability to read, write, understand, and speak English is commonly defined as low literacy levels. Computing and solving low literate patients is necessary to function in a continuous working environment and society eventually achieving everyone’s goals and expanding one’s potential and knowledge**^[35]^**. On the other hand, Health literacy is defined as the ability of an individual to adequately use their writing, reading, verbal, communication, and numerical skills, therefore positively influencing their personal health care**^[36]^**. Individuals and their families must obtain, process, and benefit from their basic constitutional rights, thus patients can understand basic health information required to take appropriate decisions to manage their health**^[37]^**.In the context of culture and civilizations, understanding health literacy gained meaningful depth. For example, this is important given the cultures, ethnicity and linguistic diversity in South Africa, the habitat to the multicultural and multilingual nation of 55 million individuals. Language, religious beliefs and customs are often each nation’s representation and perception of their culture. Eleven official languages and a variety of religions including Christianity, and traditional African religions, such as Hinduism, Judaism, Islam, and other smaller groups**^[2,38]^**.

For people like this, decision-making is led by their values. Human beings use a variety of strategies to make sure that their decisions adhere to their traditions and cultures; the success of these strategies varies depending on the patient’s mentality**^[39]^**. And when a member of a healthcare team enforces pressure on a patient or when they act on the patient’s behalf without their respective permission, the autonomy of the individual may be violated**^[40]^**.

Effective patient engagement in their personal care is necessary to improve health outcomes and treatment results, intending to ameliorate an individual’s stratification with their respective care experience, thereby reducing costs and benefit from the clinician expertise**^[41]^**. Besides this societal and community participation are commonly believed to be incremental to the development and evaluation of health services. Following the beginning of the new Sustainable Development Goals (SDG), dynamic community participation and cross-sectoral communication have emerged as a priority in health globally**^[42,43]^**.

These values can also affect the surgical care. It is commonly believed that surgical patients feel that surgery may precipitate a loss of bodily parts and therefore consider surgery to be a threat to one’s spiritual and physical body. Healthcare professionals, especially nurses due to their close communication with the patient, tend to feel uncomfortable with the provision of spiritual and religious care attributable to the deprivation of private space and deficiency in training skills to provide said religious care, especially when that spiritual care is not their professional role in the health industry. We can solve this by incorporating good communication skill trainings to health care providers.**^[44,45]^**

On the other hand, Religion affects the surgical decisions of patients. Some religious patients tend to express their refusal of certain surgical procedures due to religious beliefs. In oral and maxilla facial surgery, different graft materials can be used. Some of these materials are usually derived from porcine, bovine and sometimes human tissues (i.e., autogenous or allogenous). Some religious patients prohibit the use animal derived materials therefore requesting alternative treatment**^[46]^**. In addition, religious fasting is performed in various religions. Patients with history of metabolic/bariatric surgery (MBS) could be at risk. It may be necessary to recommend short-term avoidance of fasting in these patients who usually require intravenous fluids or develop renal calculi post-MBS. Usually following MBS, hydration with water is the main post-operative recommendation for patients**^[47]^**. Fasting patients suffer from dehydration at various rates, therefore the care team in collaboration with the patient may strategize a hydration schedule of drinking water before starting the fast and after breaking it in the evening**^[48]^**.

Disparities in surgical care can come from Socioeconomic differences. Patients with low socioeconomic status tend to present higher rates of complications and mortality.**^[49,50]^** Similarly, compared to urban residents, rural ones have lower health access like specialist doctors, blogs and magazines.**^[50–52]^**

**Figure 3.**
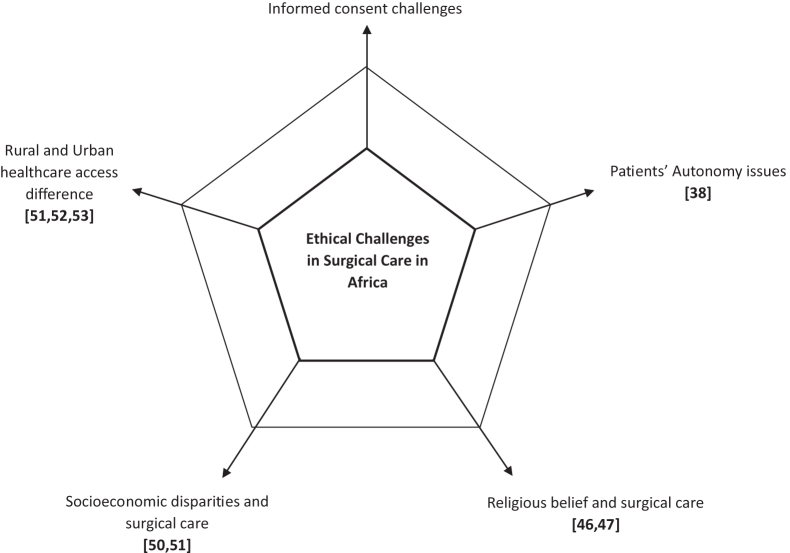
Main pillars of ethical challenges in surgical care in Africa.

Table 2 and Fig. 3 show example summary of the ethical challenges in surgical care in Africa.

**Table 2:** Example summary of ethical challenges in surgical care in Africa

Ethical challenges in surgical care in Africa	Examples	Reference(s)
Language barriers and effective communication	Leading drawbacks for IC assessment.	**^[5,6,32]^**
Literacy levels and patient understanding	Illiterate patients are incapable of understanding IC, this could increase medical errors.	**^[12,33,34]^**
Cultural perceptions of medical procedures	These perceptions are essential for the treatment outcomes.	**^[2,38]^**
Collectivistic decision-making in African cultures	Patients’ normalization tends to endure that a patient will be no worse than before.	**^[39,40]^**
Family and community involvement in healthcare decisions	Commonly believed to be incremental to the development and evaluation of health services thanks to SDG.	**^[41-43,53]^**
Refusal of certain surgical procedures due to religious beliefs	Some religious patients tend to express their refusal of certain surgical procedures.	**^[46,54]^**
Preference for alternative or traditional treatments	Some religious patients prohibit the use animal-derived grafting materials therefore requesting alternative treatment.	**^[46]^**
Impact of religious fasting on surgical care	Fasting patients suffer from dehydration, therefore collaboration between the care team and patient is necessary.	**^[47,48]^**
Socioeconomic disparities and their impact on surgical care access	Patients with low socioeconomic status tend to present higher rates of complications and mortality.	**^[49,50]^**
Differences in rural and urban healthcare access	Rural residents had lower health access when compared to urban ones.	**^[49-52]^**

## Results and discussion

This review identified a spectrum of ethical challenges inherent in surgical care across Africa, many of which are intricately connected to the continent’s cultural and religious diversity. Cultural and religious beliefs play a pivotal role in shaping patient decision-making, health-seeking behaviors, and perceptions of surgical interventions. The literature further highlights that, alongside these influences, disparities related to settlement patterns and socioeconomic status can significantly widen gaps in both access to and quality of surgical care. The following section examines how cultural and religious beliefs interact with ethical challenges and influence decision-making within African surgical settings while also proposing areas of potential recommendations.

### Cultural and religious factors influencing surgical care in Africa

Several ethical issues circumvent surgical care in Africa which are closely intertwined with its different cultures and religions. This study examined how cultural and religious beliefs affect the decision-making and health behavior of patients. It also examined the gap caused by difference in settlement and socio-economic status**^[4,55]^**.

### Recommendations for improving ethical surgical care in Africa

#### Developing culturally sensitive informed consent processes

IC should be tailored to accommodate cultural and language barriers. The healthcare providers may need to make use of translated materials, engage interpreters, and ensure patients understand the risks, benefits, and alternatives of surgery**^[20,32]^**. Otherwise, if not addressed well these may complicate the decision-making process**^[39]^**.

#### Fostering open communication and collaboration with patients and families

Through autonomy individuals have the right to decide what happens to their bodies and healthcare**^[18]^**. In African societies where collectivist decision-making prevails, healthcare providers are required to find a thin line between recognizing patient autonomy and acknowledging family and community involvement in healthcare. Patients should be included in decision-making processes by healthcare professionals, as well as, their families; hence respect their freedom of opinion and values**^[41]^**. Open channels of communication resulting in increased compliance and satisfaction can help align treatment options with individual patients’ preferences and other convictions.

#### Offering cultural competence training for healthcare professionals

In Africa, many people go to traditional healers first when they need health care services due to their deep cultural and spiritual connections. Cultural competence training must be factored into medical education as well as professional development programs to develop understanding around this area**^[14,31]^**. Healthcare providers should therefore undergo training in the sensitivity of cultural issues thereby facilitating culturally safe health care provision that leads to improved patient satisfaction**^[15]^**.

#### Advocating for equitable access to surgical care

To address these differences in surgical accessibility based on socioeconomic status, it is critical to advocate for fair resource distribution and implement creative projects that will reach the remaining underprivileged groups. This might entail collaborating with decision-makers in government, healthcare facilities, or neighborhood-based groups to bring about internal change.**^[49–51]^**

#### Engaging with religious and community leaders to address ethical concerns

Connecting with religious and community leaders may be a way to address misunderstandings pertaining to surgical care in accordance with the cultural beliefs and values of the concerned society. They are instrumental in creating links between healthcare providers and the citizens they aid**^[29,42,43]^**.

### Future research directions and potential impact on surgical care in Africa

This study focused primarily on the ethical aspects, cultural and religious perspectives, and economic factors that need to be implemented to provide high-quality surgical care in Africa. future surgical care should emphasize the development of culturally sensitive IC processes, open communication and collaboration with patients and families, improved cultural competence among healthcare providers, advocacy for equitable access to surgical care, and involvement of religious leaders and community representatives in these issues. Although this article has offered a great deal of insight, further research will be needed to fully comprehend the subject. In conclusion, studies should consider an IC strategy tailored to the cultural context of African surgical patients, assess the effect of cultural competence training on surgical outcomes, and establish models of cooperation between religious and community leaders in the delivery of surgical care. Future research is warranted to deliberate developing tactics that support moral and just surgical care throughout Africa to ameliorate these sensitive issues.

## Conclusion

Addressing ethical challenges in surgical care across Africa requires a deep understanding of the cultural and religious factors that influence patients’ decisions and behaviors. Our review shows that while the fundamental principles of medical ethics are universally important, their application in African settings is complicated by language barriers, diverse beliefs, and social inequities. To improve outcomes, it is vital for healthcare providers to adopt culturally sensitive practices, strengthen communication with patients and families, and collaborate with community leaders. Equitable access to care and ongoing training in cultural competence are also essential. Ultimately, these efforts can help ensure that surgical care in Africa is not only effective, but also respectful and inclusive of the values and needs of diverse communities.

## Data Availability

Data availability is not applicable to this article as no new data were created or analyzed in this study.
